# Impacts of Coping Mechanisms on Nursing Students’ Mental Health during COVID-19 Lockdown: A Cross-Sectional Survey

**DOI:** 10.3390/nursrep11010004

**Published:** 2021-01-12

**Authors:** Son Chae Kim, Christine Sloan, Anna Montejano, Carlota Quiban

**Affiliations:** School of Nursing, Point Loma Nazarene University, 2600 Laning Road, San Diego, CA 92106, USA; christinesloan@pointloma.edu (C.S.); amonteja@pointloma.edu (A.M.); carlotaquiban@pointloma.edu (C.Q.)

**Keywords:** COVID-19, anxiety, stress, depression, coping mechanism, resilience, spirituality, family functioning

## Abstract

The COVID-19 pandemic and consequent lockdown have precipitated significant disruption in the educational system. Nursing students are known to have higher levels of stress and anxiety than other non-nursing students, but there is a dearth of evidence regarding the impacts of the COVID-19 lockdown on their mental health and coping mechanisms. Purpose: The aim of this study was to explore the influence of coping mechanisms as predictors of stress, anxiety, and depression among nursing students during the COVID-19 lockdown. Methods: A cross-sectional online survey was conducted from 20 April to 10 May 2020 among 173 nursing students at a private university in Southern California, USA. Results: Self-reported stress, anxiety, and depression were significantly higher during the lockdown compared to the pre-lockdown period (*p* < 0.001). Almost a quarter of participants reported high stress, while more than half reported moderate-to-severe symptoms of anxiety and depression. High resilience was negatively associated with high stress (Odds Ratio (OR) = 0.46; 95% Confidence Interval (CI) = 0.22–0.98; *p* = 0.045), moderate-to-severe anxiety (OR = 0.47; 95%CI = 0.25–0.90; *p* = 0.022), and moderate-to-severe depression (OR = 0.50; 95%CI = 0.26–0.95; *p* = 0.036). Similarly, high family functioning was negatively associated with high stress (OR = 0.41; 95%CI = 0.20–0.86; *p* = 0.018), moderate-to-severe anxiety (OR = 0.41; 95%CI = 0.21–0.80; *p* = 0.009), and moderate-to-severe depression (OR = 0.41; 95%CI = 0.20–0.81; *p* = 0.011). High spiritual support was negatively associated with moderate-to-severe depression (OR = 0.48; 95%CI = 0.24–0.95; *p* = 0.035). Conclusions: During the COVID-19 lockdown, nursing students experienced remarkable levels of poor mental health. High levels of resilience and family functioning were associated with 2- to 2.4-fold lower risk of stress, anxiety, and depression, whereas high spiritual support was associated with 2-fold lower risk of depression. As the pandemic evolves, fostering these coping mechanisms may help students to maintain their psychological wellbeing.

## 1. Introduction

The COVID-19 pandemic and subsequent mandatory lockdown to suppress transmission of the SARS-CoV-2 virus have caused significant global disruption of the educational system. According to the United Nations Educational, Scientific and Cultural Organization (UNESCO), more than a billion students globally have experienced closures of educational institutions during the pandemic [1]. In California, USA, the state governor issued a statewide mandatory stay-at-home order on 19 March 2020, resulting in the shutdown of face-to-face education for students [2]. For college students, the rapid shift from in-person to online learning, as well as concerns over educational progress and future job opportunities, contributed greatly to increased levels of stress and anxiety [3,4].

Before the pandemic, a national survey of 26,181 college students in USA reported that about a half were either diagnosed or treated for anxiety, depression, or panic attacks within the past year [5]. Nursing students experienced even higher levels of stress as they adjust to challenges of rigorous academic requirements as well as clinical demands, resulting in stress-related illnesses, depression, and sleep disturbances [6,7]. In China, the arrival of the COVID-19 pandemic has resulted in even higher levels of stress, anxiety, and depression among college students [8,9]. A study from Israel demonstrated that during the third week of COVID-19 lockdown, more than half of nursing students reported moderate-to-severe anxiety symptoms [10]. The study findings showed that female gender, concerns of academic progress, and fear of infection correlated with higher anxiety.

In addition to the immediate impact of the pandemic, previous studies of earlier coronavirus outbreaks have indicated that quarantine or lockdown can result in long-term psychological consequences [11]. Anxiety symptoms and feelings of anger remained present four to six months following quarantine during the Middle East Respiratory Syndrome (MERS) outbreak in Korea [12]. Furthermore, long-term consequences of psychological distress and burnout among nurses persisted nearly two years after the original Severe Acute Respiratory Syndrome (SARS) outbreak in Canada [13].

In managing psychological distress during epidemics, various coping mechanisms appear to be effective [14]. For example, having a support group for college students quarantined at home during the earlier SARS outbreak was found to be helpful [15]. Similarly, the presence of parental support was associated with lower anxiety among college students during the COVID-19 outbreak [16]. Spiritual support also seems to be an effective coping mechanism. College students with high spiritual support had greater personal happiness and satisfaction with life, as well as better adjustment to college life [17,18,19]. Minority college students with high spiritual support employed more problem-oriented coping behaviors, such as positive reinterpretation of adverse events, resulting in lower stress and better academic performance [20]. Resilience, another coping mechanism, refers to an individual’s ability to bounce back from adversity and effectively respond to challenges [21]. Postgraduate nursing students exhibited a higher level of resilience than undergraduate nursing students, and the resilience was a positive predictor of perceived wellbeing [22]. Resilience was also positively associated with clinical communication ability and academic success [23,24]. During the COVID-19 pandemic, high resilience among nursing students was negatively associated with anxiety [10].

The psychological wellbeing of nursing students during the pandemic is critical for their academic success, and assessment of various coping mechanisms is necessary. However, there are limited studies on various coping mechanisms that enhance overall mental health of nursing students during the pandemic [10]. The aim of this study was to explore the influence of coping mechanisms as predictors of stress, anxiety, and depression among nursing students during the COVID-19 pandemic and subsequent lockdown.

## 2. Methods

### 2.1. Design and Sample

This cross-sectional study was conducted using an online survey platform Qualtrics XM (Provo, UT, USA) during the COVID-19 pandemic lockdown. All undergraduate and graduate nursing students enrolled in the spring semester 2020 at a private university in Southern California, USA were eligible to participate in the study.

### 2.2. Study Questionnaire

The study survey included valid and reliable instruments that measure resilience, spiritual support, family functioning, stress, anxiety, and depression. The respondents were asked to assess their current mental health status as well as to estimate their pre-lockdown mental health. Demographic information, including age, gender, ethnicity, and nursing program enrollment, was also collected.

The Connor–Davidson Resilience Scale (CD-RISC)-10 asks respondents to assess their adaptability in challenging situations and the ability to bounce back [25]. The response options on a 5-point Likert scale range from 0 (not true at all) to 4 (true all the time). The possible maximal score is 40, with a higher score indicating higher resilience. The Cronbach’s alpha was previously reported as 0.92 and was 0.83 in this study.

The 12-item Spirituality Support Scale evaluates respondents’ perceived spiritual support from a higher power, religious faith, or beliefs on a 4-point Likert scale ranging from 1 (strongly disagree) to 4 (strongly agree) [26]. The summation scores range from 12 to 48, with a higher score indicating higher spiritual support. The Cronbach’s alpha was previously reported as 0.97 and was 0.96 in this study.

The Family APGAR questionnaire measures satisfaction with support from family members [27]. It includes five indicators of family functioning: adaptation, partnership, growth, affection, and resolve, on a 3-point Likert scale ranging from 0 (hardly ever) to 2 (almost always). Scores of 8–10 indicate a highly functional family, while scores of 4–7 and 0–3 indicate moderate and severely dysfunctional families, respectively. The inter-item correlation coefficients were reported to be 0.63 to 0.71.

The Perceived Stress Scale (PSS) assesses respondents’ perception of stress by eliciting thoughts and feelings during the past month [28]. It consists of 10 items in two subscales, including 6-item positive and 4-item negative factors on a 5-point Likert scale ranging from 0 (never) to 4 (very often). The responses of four items in the negative factor were reversed with possible total scores ranging from 0 to 40. Scores of 0–13, 14–26, and 27–40 were assessed as low, moderate, and high stress, respectively. Cronbach’s alpha was previously reported as 0.83, and was 0.85 in this study.

The General Anxiety Disorder-7 (GAD-7) and Patient Health Questionnaire-9 (PHQ-9) are widely used tools that assess the symptoms of anxiety and depression, respectively [29]. The response options range from 0 (not at all) to 3 (nearly every day). Scores ≥10 are considered moderate-to-severe anxiety or depression that potentially warrant further follow-up. The sensitivity and specificity of the GAD-7 for anxiety disorder were 72% and 80%, respectively. Similarly, the sensitivity and specificity of the PHQ-9 for the major depressive disorder were 88% and 88%, respectively.

### 2.3. Data Collection Procedures

The Institutional Review Board of the university approved this study (PLNU IRB ID#17877). Recruitment emails containing a hyperlink to the online survey were sent to all nursing students enrolled in the spring semester 2020. Written informed consent was waived due to minimal risk associated with participation. Students were assured that their participation in the study was confidential and voluntary. They were also reminded that participation or lack thereof would not affect their course grades or relationship with the school. Completion of the online survey indicated their consent to the study. Data were collected from 20 April to 10 May 2020, and at study closure, a $10 gift card was given to 10 randomly selected students. This study was carried out following the rules of the Declaration of Helsinki.

### 2.4. Data Analysis

In this study, poor mental health was defined using the symptom severity cutoff scores for high stress (PSS scores ≥ 27), moderate-to-severe anxiety (GAD-7 scores ≥ 10), and moderate-to-severe depression (PHQ-9 scores ≥ 10). Coping mechanisms were categorized into dichotomous variables using the following cutoff scores: CD-RISC-10 scores ≥ median of 29 (high resilience); Spirituality Support Scale scores ≥ median of 36 (high spiritual support); and family APGAR scores ≥ 8 (high family functioning).

Descriptive statistics summarized sample characteristics and key study variables. Wilcoxon signed-rank tests were performed to compare the median scores of high stress, moderate-to-severe anxiety, and moderate-to-severe depression before and during the lockdown. Kendall’s tau correlation procedures were performed to explore potential correlations between the dichotomous variables of poor mental health, coping mechanisms, and demographic variables. Statistically significant demographic variables and coping mechanisms were entered into multivariate logistic regression models to explore the influence of coping mechanisms as predictors of poor mental health. All statistical analyses were performed using SPSS software, version 26.0 (IBM Corporation, Armonk, NY, USA). The level of significance level was set at *p*-value < 0.05, and all tests were two-tailed.

## 3. Results

### 3.1. Sample Characteristics

Of 447 students invited, 173 completed the survey and were included in the statistical analysis (38.7% participation rate). The average age was 25 years old, and most were female (93.1%). More than half were white (57.8%) and pre-licensure undergraduate students (76.8%). Almost a quarter (23.1%) were registered nurses enrolled in graduate programs or a baccalaureate degree completion program. About 14 percent had worked with COVID-19 patients, while three quarters had experienced quarantine or self-isolation during the pandemic (75.7%). For coping mechanisms, the median (IQR) scores of resilience, spiritual support, and family functioning were 29 (25, 32), 36 (33, 43), and 9 (6.5, 10), respectively.

### 3.2. Mental Health before and during COVID-19 Lockdown

**Figure 1:**
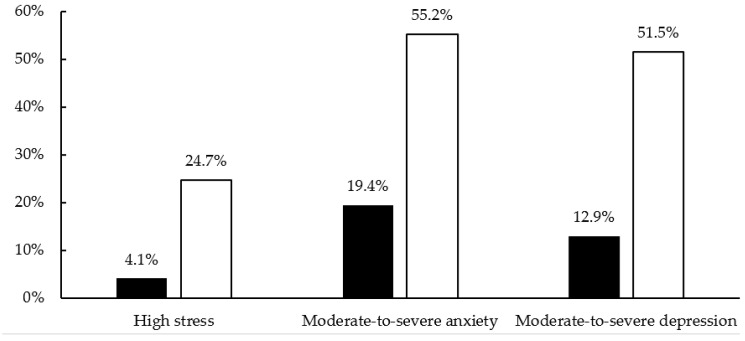
Poor mental health status before and during COVID-19 lockdown (*N* = 173). Black bars and white bars represent the poor mental health status before and during the COVID-19 lockdown, respectively. High stress = Perceived Stress Scale scores ≥ 27; moderate-to-severe anxiety = GAD-7 scores ≥ 10; moderate-to-severe depression = PHQ-9 scores ≥ 10.

Compared with the pre-lockdown period, students reported higher stress (median [IQR]; 16 [13, 18] vs. 23 [18, 26.3]; *p* < 0.001), moderate-to-severe anxiety (median [IQR]; 4 [2, 8.3] vs. 10 [5, 15]; *p* < 0.001), and moderate-to-severe depression (median [IQR]; 3 [1, 6] vs. 10 [4, 15]; *p* < 0.001) during the lockdown. Similarly, more students experienced high stress (4.1% vs. 24.7%), moderate-to-severe anxiety (19.4% vs. 55.2%), and moderate-to-severe depression (12.9% vs. 51.5%) during the lockdown (Figure 1).

### 3.3. Predictors of Poor Mental Health

Table 1 shows bivariate Kendall’s tau correlations between coping mechanisms and poor mental health. High resilience and high family functioning were negatively correlated with high stress, moderate-to-severe anxiety, and moderate-to-severe depression (*p* < 0.05). Similarly, there were significant negative correlations between high spiritual support and anxiety and depression. By contrast, none of the demographic variables correlated significantly with high stress, moderate-to-severe anxiety, or moderate-to-severe depression.

The results of multivariate logistic regression procedures are shown in Table 2. High resilience was a significant predictor of lower risks of high stress (Odds Ratios (OR) = 0.46; 95% Confidence Interval (CI) = 0.22–0.98; *p* = 0.045), moderate-to-severe anxiety (OR = 0.47; 95%CI = 0.25–0.90; *p* = 0.022), and moderate-to-severe depression (OR = 0.50; 95%CI = 0.26–0.95; *p* = 0.036). Similarly, high family functioning was also a significant predictor of lower risks of high stress (OR = 0.41; 95%CI = 0.20–0.86; *p* = 0.018), moderate-to-severe anxiety (OR = 0.41; 95%CI = 0.21–0.80; *p* = 0.009), and moderate-to-severe depression (OR = 0.41; 95%CI = 0.20–0.81; *p* = 0.011). In addition, high spiritual support was a significant predictor of lower risk of depression (OR = 0.48; 95%CI = 0.24–0.95; *p* = 0.035).

## 4. Discussion

It is remarkable how much the COVID-19 lockdown has impacted the psychological wellbeing of nursing students. The proportion of students with high stress level during the lockdown jumped 6-fold compared to the pre-lockdown period, while moderate-to-severe anxiety and depression increased nearly 3–4-fold. Interestingly, psychological distress appears to be ameliorated by potentially modifiable factors, such as resilience, family functioning, and spiritual support. In multivariate analyses, high resilience and family functioning were independently associated with a 2- to 2.4-fold lower risk of high stress, moderate-to-severe anxiety, and moderate-to-severe depression. In addition, high spiritual support was also independently associated with 2-fold lower risk of moderate-to-severe depression. By contrast, demographic variables, such as age, educational background, or ethnicity, showed no correlation with poor mental health during the lockdown.

It is plausible that resilience, family functioning, and spiritual support among nursing students can be nurtured so that they can better manage unexpected challenges impacting psychological equilibrium. For example, a randomized controlled trial (RCT) of resilience training based on cognitive behavior therapy among newly licensed registered nurses showed significant decreases in stress, anxiety, and depression [30,31]. Perhaps, resilience and spiritual support are complimentary coping mechanisms. Spiritual support arises from a sense of connection to an external higher power, whereas resilience is one’s internal capacity to bounce back and adapt to stressful circumstances [32,33,34,35]. In our study, about half of the students reported moderate-to-severe anxiety and depression during the lockdown, which is consistent with a study of nursing students in Israel that reported more than half with anxiety [10]. However, other dimensions of psychological distress, such as perceived stress and depression, were not assessed in this previous study. Another study among university students and associates in Germany also showed higher levels of health anxiety during the COVID-19 pandemic compared to pre-pandemic [36]. High family function as a protective factor against poor mental health is consistent with another previous study. Among college students in China, lower anxiety symptoms were associated with living with parents during the COVID-19 pandemic [16]. Although these authors did not assess family functioning as a coping mechanism, it is likely that simply living with parents during isolation reduces symptoms of anxiety and depression among college students.

### 4.1. Implications for Nursing Education

The COVID-19 pandemic has caused significant disruption in education worldwide. Although most colleges have shifted to online learning, nursing schools must continue to have clinical practicums, which require students to work in close contact with patients. The concern of viral transmission and the unpredictable progression of the COVID-19 pandemic may further increase students’ uncertainty of academic advancement and affect their mental health negatively. Therefore, it is imperative for nurse educators to help students to identify and develop optimal coping strategies to minimize anxiety due to the pandemic. Providing clear guidelines for infection control will help students to feel confident about their safety and minimize stress as they work with patients. Furthermore, it is critical to provide a supportive learning environment that helps students to develop effective coping strategies [32]. For example, nursing school can develop and integrate a resilience-building program throughout the nursing curriculum to assist students in managing stress associated with academic, social, and personal challenges. Such a resilience-building program may include mindfulness-based stress reduction strategies, muscle relaxation exercise, self-care, communication skills, problem-solving skills, or study skills. These activities may be implemented via lectures, reflective journaling, experience sharing, role-playing, or homework assignments [33]. Studies have shown that such programs can help students to utilize effective coping strategies and time management, which improves mental health and academic success [33,34,35,37]. In addition, tailored resilience interventions based on needs assessment and skill-building activities may be helpful [36]. Given the need for social distancing, online platforms may also be useful for spiritual and peer support, as well as resilience training to enhance psychological wellbeing [15,38,39].

### 4.2. Limitations

There are several limitations to this study. Because this was a cross-sectional study, the resilience, family functioning, and spiritual support as predictors of mental health should not be taken as a cause-and-effect relationship. Such a causal relationship can be determined only through interventional studies. Second, less than 40% of the enrolled students completed the survey, which is not unusual for an online survey. However, this may have introduced a selection bias. Third, the self-reported data collection method could have over- or underestimated the symptoms of stress, anxiety, and depression. Fourth, pre-lockdown data were collected retrospectively, which may have introduced recall bias. Fifth, this study was conducted in spring 2020 during the initial wave of the COVID-19 pandemic in the US. Thereafter, the pandemic has evolved rapidly with subsequent case spikes, as well as the advent of improved treatments and vaccines. Therefore, the study findings may be most applicable to the initial phase of such pandemics. Finally, the study findings are based on a relatively small sample size from a single institution and may not be broadly generalizable. Future interventional studies are needed to confirm these study findings as well as to determine the effectiveness of fostering coping mechanisms among nursing students.

## 5. Conclusions

During the COVID-19 lockdown, nursing students experienced remarkable levels of poor mental health. This study showed that high resilience, family functioning, and spiritual support were predictors of lower stress, anxiety, and depression. As the pandemic evolves, fostering these coping mechanisms may help students to maintain their psychological wellbeing.

## Figures and Tables

**Table 1:** Correlations with poor mental health during COVID-19 lockdown (*N* = 173).

	High Stress (*r*)	Moderate-to-Severe Anxiety (*r*)	Moderate-to-Severe Depression (*r*)
Age	−0.07	−0.14	−0.09
Female	0.09	0.01	0.03
Pre-licensure undergraduate students	0.11	0.02	0.06
Quarantine or self-isolation experience	0.10	0.06	−0.03
High resilience ^a^	−0.20 *	−0.22 **	−0.23 **
High family functioning ^b^	−0.22 **	−0.24 **	−0.28 ***
High spiritual support ^c^	−0.08	−0.21 *	−0.24 **

* p < 0.05; ** *p* < 0.01; *** *p* < 0.001 by Kendall’s Tau test. High stress = Perceived Stress Scale scores ≥ 27; moderate-to-severe anxiety = GAD-7 scores ≥ 10; moderate-to-severe depression = PHQ-9 scores ≥ 10. ^a^ CD-RISC-10 scores ≥ 29; ^b^ Family APGAR scores ≥ 8; ^c^ Spirituality Support Scale scores ≥ 36.

**Table 2:** Predictors of poor mental health during COVID-19 lockdown (*N* = 173).

	OR	95%CI	*p*-Value
**High stress**			
High resilience ^a^	0.46	0.22–0.98	0.045
High family functioning ^b^	0.41	0.20–0.86	0.018
**Moderate-to-severe anxiety**			
High resilience ^a^	0.47	0.25–0.90	0.022
High family functioning ^b^	0.41	0.21–0.80	0.009
**Moderate-to-severe depression**			
High resilience ^a^	0.50	0.26–0.95	0.036
High family functioning ^b^	0.41	0.20–0.81	0.011
High spiritual support ^c^	0.48	0.24–0.95	0.035

OR = odds ratio; CI = confidence interval. High stress = Perceived Stress Scale scores ≥ 27; moderate-to-severe anxiety = GAD-7 scores ≥ 10; moderate-to-severe depression = PHQ-9 scores ≥ 10; ^a^ CD-RISC-10 scores ≥ 29; ^b^ Family APGAR scores ≥ 8; ^c^ Spirituality Support Scale scores ≥ 36.

## Data Availability

The data presented in this study are available on request from the corresponding author. The data are not publically available due to ethical considerations.
